# Stromal Expression of Decorin, Semaphorin6D, SPARC, Sprouty1 and Tsukushi in Developing Prostate and Decreased Levels of Decorin in Prostate Cancer

**DOI:** 10.1371/journal.pone.0042516

**Published:** 2012-08-03

**Authors:** Alexander Henke, O. Cathal Grace, George R. Ashley, Grant D. Stewart, Antony C. P. Riddick, Henry Yeun, Marie O’Donnell, Richard A. Anderson, Axel A. Thomson

**Affiliations:** 1 Medical Research Council, Centre for Reproductive Health, The Queens’s Medical Research Institute, Edinburgh, Scotland, United Kingdom; 2 Edinburgh Urological Cancer Group, Division of Pathology, Institute of Genetics and Molecular Medicine, University of Edinburgh, Edinburgh, United Kingdom; IIT Research Institute, United States of America

## Abstract

**Background and Aim:**

During prostate development, mesenchymal-epithelial interactions regulate organ growth and differentiation. In adult prostate, stromal-epithelial interactions are important for tissue homeostasis and also play a significant role in prostate cancer. In this study we have identified molecules that show a mesenchymal expression pattern in the developing prostate, and one of these showed reduced expression in prostate cancer stroma.

**Methodology and Principal Findings:**

Five candidate molecules identified by transcript profiling of developmental prostate mesenchyme were selected using a wholemount in situ hybridisation screen and studied Decorin (Dcn), Semaphorin6D (Sema6D), SPARC/Osteonectin (SPARC), Sprouty1 (Spry-1) and Tsukushi (Tsku). Expression in rat tissues was evaluated using wholemount in situ hybridisation (postnatal day (P) 0.5) and immunohistochemistry (embryonic day (E) E17.5, E19.5; P0.5; P6; 28 & adult). Four candidates (Decorin, SPARC, Spry-1, Tsukushi) were immunolocalised in human foetal prostate (weeks 14, 16, 19) and expression of Decorin was evaluated on a human prostate cancer tissue microarray. In embryonic and perinatal rats Decorin, Semaphorin6D, SPARC, Spry-1 and Tsukushi were expressed with varying distribution patterns throughout the mesenchyme at E17.5, E19.5, P0.5 and P6.5. In P28 and adult prostates there was either a decrease in the expression (Semaphorin6D) or a switch to epithelial expression of SPARC, and Spry-1, whereas Decorin and Tsukushi were specific to mesenchyme/stroma at all ages. Expression of Decorin, SPARC, Spry-1 and Tsukushi in human foetal prostates paralleled that in rat. Decorin showed mesenchymal and stromal-specific expression at all ages and was further examined in prostate cancer, where stromal expression was significantly reduced compared with non-malignant prostate.

**Conclusion and Significance:**

We describe the spatio-temporal expression of Decorin, Semaphorin6D, SPARC, Spry-1 and Tsukushi in developing prostate and observed similar mesenchymal expression patterns in rat and human. Additionally, Decorin showed reduced expression in prostate cancer stroma compared to non-malignant prostate stroma.

## Introduction

Mesenchymal-epithelial interactions, mediated via cell-cell signalling, play a crucial role in specification of mammalian organs such as the prostate, kidney, lung, and mammary gland and also in tissue homeostasis of adult tissues. Mesenchyme is the embryonic precursor of adult stroma. In males the prostate differentiates and grows from the urogenital sinus and is regulated by testicular androgens. In females, the urogenital sinus develops into the uterus and vagina, though it will form a prostate if exogenous androgens are administered. Androgen effects are mediated via the androgen receptor (AR) and studies in rodent models have demonstrated that AR is expressed initially in the mesenchyme and subsequently in the epithelium of the developing prostate. Tissue recombination experiments using mesenchyme and epithelium from either wild type or AR-deficient mice revealed that a prostate can only develop when the mesenchyme has a functional AR, while epithelial AR is not required [1]–[3]. The concept of embryonic mesenchymal and adult stromal cells as mediators of organ-specificity was underlined by a study that demonstrated diversity and positional memory in adult human fibroblasts [4]. In the normal human adult prostate the majority of the stromal compartment is made up of smooth muscle cells and fibroblasts, while the remainder consists of endothelial cells, pericytes, lymphocytes and macrophages [5].

Recent studies have highlighted a role for the stromal compartment in regulation of cancer cell progression, which is now generally accepted [6]. Within the tumour microenvironment cancer-associated fibroblasts (CAF) have been proposed as a key source of pro-tumourigenic paracrine mediators [7]–[9]. TGFβ1 is one of the factors secreted by cancer-associated fibroblasts (CAF) and can act in an autocrine or paracrine loop by binding to the receptor complex TGFβRI/II on stromal and/or epithelial cells in breast and prostate cancer cells [10]. The pro-tumourigenic potential of TGFβ1 was shown when the genetic ablation of the TGFβRII in fibroblasts resulted in reduced tumour growth in a mouse model [10], [11]. Another important aspect of stromal pro-tumourigenic activity is the heterogeneity of fibroblast subpopulations and the lack of appropriate markers for them. Several CAF markers have been proposed but identification of distinct subpopulations co-expressing some of them and the contribution of these subpopulations to tumour growth is recently emerging [12]–[14].

There is a clear relationship between developmental signalling and disease. In the Dunning rat model of prostate cancer, the inclusion of embryonic mesenchyme reduced tumour growth, underlining the instructive power of mesenchymal cells on malignant cells [15]. Additionally, the McNeal hypothesis suggests that developmental pathways are re-activated in prostatic disease [16].

Thus, the identification of mesenchymal/stromal mediators of mesenchymal-epithelial communication is paramount for the understanding of development and disease of an organ. Secreted or membrane-bound proteins appear to be the most likely mediators of stromal/epithelial paracrine signalling.

In a previous study from our group, serial analysis of gene expression (SAGE) of mesenchyme from the neonatal rat urogenital tract identified 219 putative ‘mesenchymal’ transcripts that were expressed at higher levels in mesenchyme than adjacent epithelial tissue. This list might contain candidates for paracrine mediators of mesenchymal-epithelial dialogue [17]. Within the 219 candidate transcripts we identified a subset that encoded secreted or membrane-bound molecules, which are the most likely candidates for mesenchymal-epithelial interaction. To date, we have analysed and validated the expression of some of these candidates in prostate development. We demonstrated that Dlk1 and Notch2 signalling was important for branching as well as epithelial and smooth muscle differentiation in prostate organ culture systems [18]. The receptor EphB3 and its ligand EphrinB1 were also detected in prostate mesenchyme, and organ culture with EphrinB1-Fc ligand resulted in increased organ area but decreased budding with enlarged bud tips, while incubation with EphB3-Fc receptor reduced organ size and reduced budding [19]. Pleiotrophin (Ptn) was found to be expressed in developing prostate mesenchyme and it regulated the growth of developing mesenchyme, epithelium and CAFs. Additionally, androgen signalling increased Ptn expression [20].

Taken together, these studies suggest that a high proportion of the candidates identified by our SAGE profiling play a role in prostate development and disease. In the current study, we have further analysed candidate molecules from our earlier SAGE studies to identify those expressed in rat and human prostate development, and in prostate cancer. Our rationale was that identification of mesenchymally expressed molecules would give a better understanding of stromal biology. We selected secreted and membrane bound molecules identified in our SAGE studies and performed a wholemount in situ hybridisation screen (WISH) to define whether they showed mesenchymal expression.

Here we show five candidates identified by WISH screen that were confirmed as showing mesenchymal expression: *Decorin, Semaphorin6D, SPARC, Spry-1* and *Tsukushi* (Table 1). These were examined during rat and human prostate development via immunohistochemistry.

**Table 1:** Overview of candidate genes.

Official gene symbol (rat)	Official gene name	Other names	Accession numbers/Uni gene code	Protein function
Dcn	Decorin	bone proteoglycan II; PG40; PG-S2; dermatan sulfate proteoglycan-II(DSPG)	NM_024129.1/Rn.106103	ECM protein; binds the N-terminal region of collagen VI; involved in kidney and lung branching; potential tumour suppressor [29], [32], [47], [48]
Sema6D	Semaphorin6D	–	NM_001107768.1/Rn.8257	Family of Semaphorin axon guidance molecules [35] but Sema6D is involved in heart development [49]
SPARC	secreted protein,acidic, cysteine-rich	Osteonectin (ON); basement-membrane protein 40(BM-40)	NM_012656/Rn.98989	Secreted structural protein involved in branching of lung [50]and mesonephros [51]; suppresses inflammation in ovarian cancer tumour microenvironment; tumour-suppressive in Tramp mouse model of PCa [52]
Spry-1	Sprouty homologue 1	–	NM_001106427.1/Rn.22787	FGF antagonist; crucial for uretic bud branching for kidney formation and also involved in prostate cancer [53]–[56]
Tsku	Tsukushi	hepatic protein EIIH; leucine-richrepeat-containing protein 54; earlyinsulin-induced hepaticgene protein	NM_001009965/Rn.8672	antagonizes FGF and BMP signalling in chicken and Xenopus early neuronal embryonic development[57]–[60]; Tsku gene knock out has small brain andlacks commissure [61]

Decorin and Tsukushi were the only candidates with stromal-only expression and were subsequently investigated in prostate cancer (PCa) tissues. Decorin showed a significant downregulation in comparison to non-malignant tissues, while Tsukushi expression did not show any differential expression.

## Results

### Identification and Confirmation of Mesenchymally Expressed Transcripts

**Figure 1:**
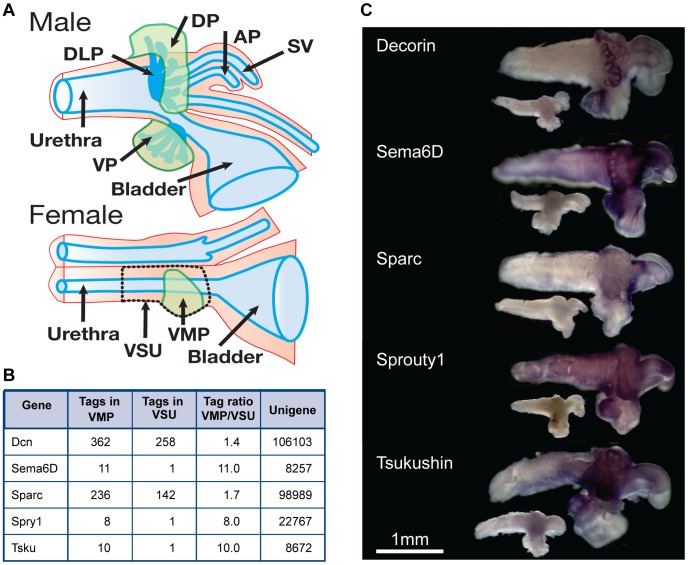
Rat urogenital tract (UGT) anatomy and the expression *of Decorin, Tsukushi, Semaphorin6D, SPARC*, and *Sprouty1* in mesenchyme. A) Schematic overview of rat UGT anatomy at perinatal stage (P0.5) for the male (top) and female (bottom). The green shaded area corresponds to inductive mesenchyme, which is found in both, male and female UGTs. The female VMP mesenchyme was used for SAGE gene profiling studies, and our WISH screen aimed to identify those transcripts expressed in male prostate mesenchyme. Figure based on Thomson, 2001 [46]. B) Transcript expression levels of *Decorin, Sema6D, SPARC, Spry-1* and *Tsku* obtained via SAGE profiling of P0.5 female rat VMP [17]. Transcripts were identified via short, transcript-specific sequences called tags and their count reflects the transcript expression level. The ratio between VMP and VSU tag counts represents the likelihood of restriction to the inductive mesenchyme; values at or over 1.4 suggested mesenchyme-specific expression. C) Detection of *Decorin, Sema6D, SPARC, Spry-1* and *Tsku* mRNA distribution via in situ hybridisation in male rat P0.5 UGTs. The small insets show UGTs treated with the negative control sense RNA probes, scale bar is 1 mm Abbreviations: AP anterior prostate, DP dorsal prostate, DLP dorsolateral prostate, VP ventral prostate, VMP ventral mesenchymal pad, VSU; VMP + smooth muscle + urothelium, SV seminal vesicle.

In a previous study from our laboratory, SAGE analysis of rat prostate mesenchyme identified 219 transcripts that were expressed in inductive mesenchyme; these were promising potential regulators of organogenesis. Our SAGE profiling studies used the female prostate anlagen, termed VMP, since it lacked epithelia and our goal was to identify non-epithelial transcripts. Fig. 1A shows the homology between the mesenchyme of female and male urogenital tract (UGT) at P0.5, and the important subregions of mesenchyme are shaded in green. These SAGE studies identified transcripts likely to be expressed within the mesenchyme [17]. Here, we focused on secreted or membrane-bound molecules within the 219 transcript list as the most logical candidates for mesenchymal-to-epithelial signalling. To define which of our candidate molecules showed mesenchymal/stromal specific expression patterns, a small WISH screen was performed. Five candidates *Decorin, Semaphorin6D, SPARC, Spry-1* and *Tsukushi* were confirmed as mesenchyme-expressed in this screen and chosen for further investigation (Figure 1B, Table 1). Fig. 1B illustrates the numbers of transcripts (identified via “tags” from the 3′ end of the mRNA), normalised per million tags to account for different tag counts between libraries), which gave an approximation of transcript expression level. From our list of five candidates, *Decorin* and *SPARC* were the most highly expressed transcripts, and then *Sema6D, Spry-1* and *Tsku*. Transcript levels were measured in two different libraries (VMP and VSU) and the ratio between these gave an indication of the likelihood of localisation to the VMP mesenchyme, as the VMP is a subset of the VSU [17]. Molecules expressed ubiquitously gave ratios closer to 1, while those showing VMP enrichment had ratios of 1.4 or greater.

Next, we performed WISH on rat neonatal urogenital tracts (UGT) to determine the expression of mRNAs encoding our five candidate molecules (Fig. 1C). *Decorin* and *SPARC* transcripts showed a highly restricted expression pattern in mesenchyme of the prostate (all lobes), while *Sema6D*, *Sprouty1* and *Tsukushi* were expressed in prostate mesenchyme as well as urethral mesenchyme. In general, distinct expression was visible in mesenchyme adjacent to epithelial ducts and/or the wider epithelium-surrounding mesenchyme in the ventral prostate (VP), the dorsal prostate (DP), and the dorsolateral prostate (DLP). *Decorin* showed the most restricted expression pattern in prostate mesenchyme (Fig. 1C) and was clearly visible in mesenchyme adjacent to epithelial buds, and was absent from non-prostatic mesenchyme such as the urethral mesenchyme.

### Immunolocalisation of Decorin in Developing Rat Prostatic Mesenchyme

**Figure 2:**
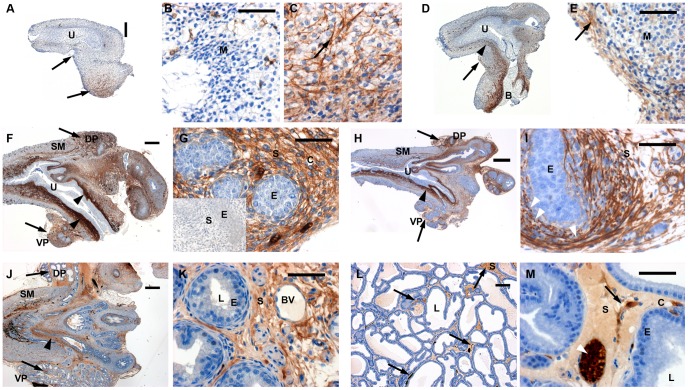
Immunolocalisation of Decorin in the developing rat prostate. Developmental stages of E17.5 (A-C), E19.5 (D, E), P0.5 (F, G), P6.5 (H, I), P28 (J, K) and young adult (L, M) were investigated. At developmental stages E17.5, E19.5 the magnifications show the areas of prospective VP, while at P0.5 and P6.5 the VP or DP are shown. Decorin was absent in epithelium and the smooth muscle compartment but present in the mesenchyme/stroma. The expression was especially strong in mesenchyme adjacent to urethral and prostatic epithelium, as indicated by black arrowheads and arrows. Notably, Decorin appeared to co-localise with structural fibres in the extracellular matrix, indicated by white arrowheads in I and M. Legend: B bladder, BV blood vessel, C capillary, DP dorsal prostate, E epithelium, M mesenchyme, L lumen of prostatic ducts, S stroma, SM smooth muscle, U urethra, VP ventral prostate. Scale bars: 200 µm in A, D, F, H and L, 400 µm in J, and 50 µm in all others. Please note that panel H has been size adjusted – including the scale bar - and is not at the same magnification as panels A, D and F.

We next examined the distribution of Decorin protein to ascertain whether it showed a similar distribution to the mRNA, and if it was also restricted to the mesenchyme. At E17.5 Decorin was expressed in a subset of the UGT mesenchyme (Fig. 2A); the bladder and adjacent to the prospective VP (Fig. 2B), the expression was restricted to single cells with weak expression in the cytoplasm and a strong signal at the cell surface. At E19.5 Decorin showed strong expression in the prostatic mesenchyme and weaker staining around bladder with “hot spots” (Fig. 2D). However, the prospective VP and DP also showed Decorin expression but not as strong as that in the peri-urethral mesenchyme (Fig. 2D, E). Neonatal P0.5 and P6.5 UGTs showed similar staining with mesenchyme-only expression and a complete absence in both urethral and prostatic epithelium (Fig. 2F–I). Interestingly, this expression pattern differed from the WISH pattern, where Decorin was found exclusively in mesenchyme within prostatic lobes but not the peri-urethral area, and may reflect secretion and diffusion of Decorin protein. At day 28, Decorin expression was found in the stroma surrounding prostatic ducts, and was absent from epithelia (Fig. 2J, K). At adulthood the VP was morphologically fully differentiated, containing Decorin-negative epithelia, and Decorin-positive stroma (Fig. 2L, M). In summary, Decorin showed mesenchyme and stromal-specific expression at all investigated developmental stages and a complete absence in the epithelium. Furthermore, it was localised mainly to the acellular interstitium.

### Immunolocalisation of Tsku in Developing Rat Prostatic Mesenchyme

**Figure 3:**
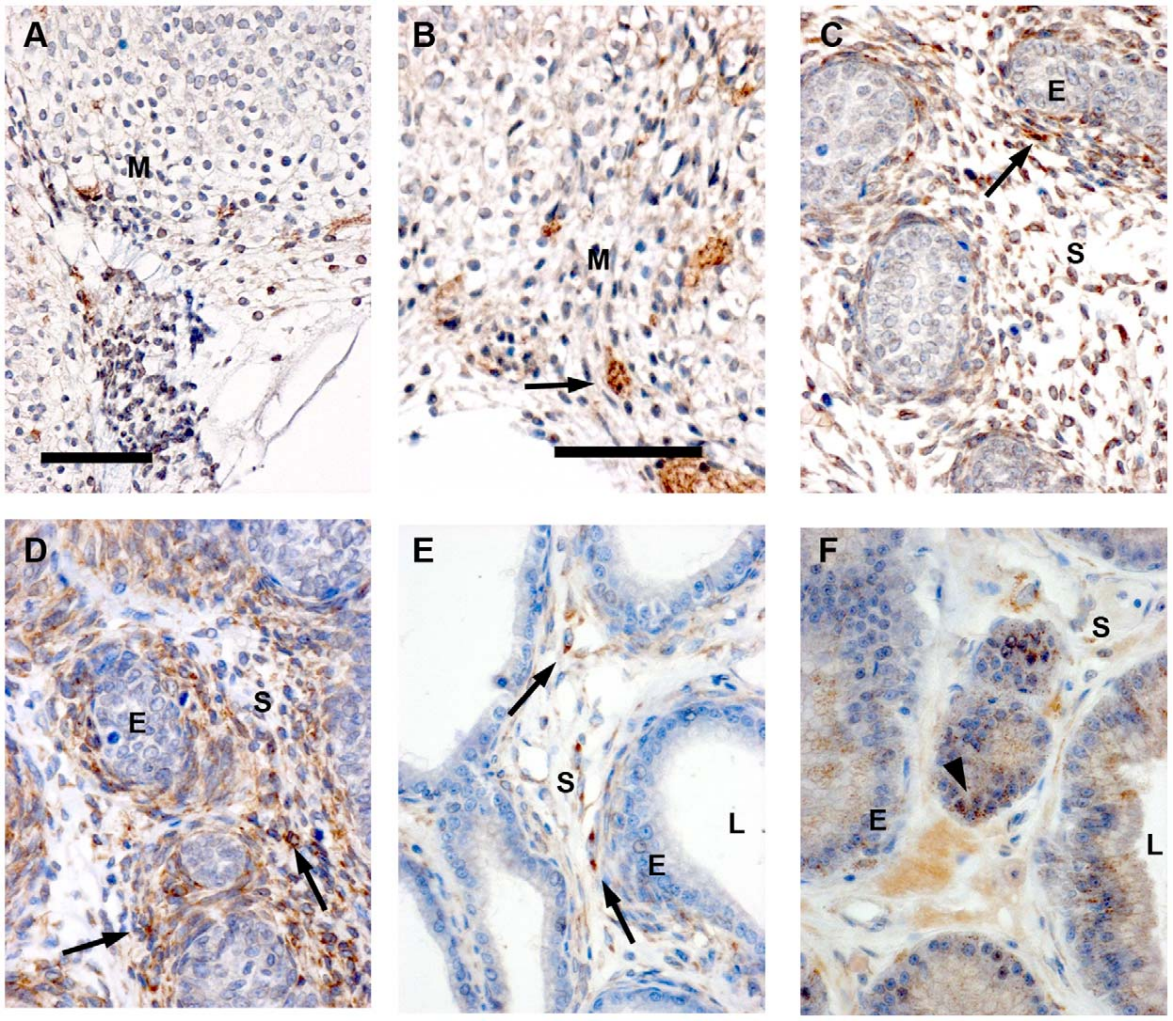
Immunolocalisation of Tsukushi in the developing rat prostate. Developmental stages of E17.5 (A), E19.5 (B), P0.5 (C), P6.5 (D), P28 (E) and young adult VP (F) were investigated. At developmental stages E17.5, E19.5 the magnifications show the areas of prospective VP, while at P0.5 and P6.5 it is either the VP or the DP. Tsku protein expression was mesenchymal/stromal only until P28 (arrows). However, in adult VP, there was additional low expression in the epithelium (arrowhead in F). Interestingly, smooth muscle cells were also positive for Tsku (P0.5 to P28). Scale bars equal 200 µm.

Tsku expression at E17.5 and E19.5 was confined to mesenchyme and smooth muscle, and showed a cytoplasmic localisation in the VP and DP (Fig. 3A, B). At P0.5 and P6.5, Tsku was absent in the epithelia but present in the smooth muscle and mesenchyme that surrounds epithelial ducts in the VP and DP (Fig. 3 C, D). The *Tsku* mRNA and protein showed similar distribution patterns.

At day 28, the epithelium lacked Tsku and stromal expression was less intense (Fig. 3 E). The fully differentiated adult VP showed a further decrease in staining compared to P28. Additionally, Tsku was observed in epithelial cells (Fig. 3 F).

### Immunolocalisation of Semaphorin6D, SPARC and Spry-1 in Developing Rat Prostatic Mesenchyme

Semaphorin6D was expressed in the mesenchyme, including the VP anlagen, at E17.5 to E19.5 (supplementary Fig. S1A, B), perinatal VP and DP lobes, and at P6.5 UGTs but was absent in the epithelia (Fig. S1 C, D). Surprisingly, immunostaining was confined to the nuclei although Semaphorins are usually reported to be localised at the plasma membrane. Nevertheless, WISH pattern and protein detection were consistent in P0.5 UGTs. However, in fully differentiated adult VP, only leukocytes were found to express Semaphorin6D (Fig. S1F). In summary, the Semaphorin6D expression was strongest and most consistent in the mesenchyme of E17.5 to P0.5 UGTs with nuclear localisation.

Immunostaining of SPARC was not detectable in E17.5 or E19.5 old UGTs (data not shown). Weak mesenchymal expression in the VP and DP was observed at P0.5 and was absent from the epithelium (Fig. S2A). SPARC was expressed in the mesenchyme surrounding epithelial buds at both P0.5 and P6.5 but its staining was more prevalent and intense in the latter stage. In contrast, there was no epithelial SPARC expression detectable (Fig. S2B). The patterns for *SPARC* detection via WISH and immunohistochemistry (IHC) were consistent in P0.5 UGTs.

At P28 and adult, SPARC expression was very low in the stroma at P28 (Fig. S2C) and absent in adult VP (Fig. S2D). However, there was a strong expression in a subset of epithelial luminal cells, localised to the nuclei. In summary, while absent in embryonic and foetal stages, SPARC was expressed in the mesenchyme during the branching period but there is a stromal-epithelial reversal of expression during the formation and maintenance of adult prostate with strong expression in some epithelial cells but not the stroma.

Rat Spry-1 protein was detected in the mesenchyme in developing VP in E17.5 and E19.5 UGTs (Fig. S3A, B). At perinatal stage P0.5 (Fig. S3C), the Spry-1 detection was strongest in the mesenchyme adjacent to epithelial ducts and less intense in mesenchymal cells between ducts, and it was localised adjacent to the nucleus rather than being distributed in the cytoplasm (Fig. S3F). The patterns for Spry-1 detection via WISH and IHC were comparable in P0.5 UGTs (Fig. S3C).

There was weak detection of Spry-1 within prostatic epithelial ducts at P0.5 and was more pronounced at P6.5, while mesenchymal Spry-1 expression remained unaltered (Fig. S3D). In contrast, prostatic lobes at day 28 and adult VP showed a reversal of Spry-1 expression compared with P0.5 UGTs. In the differentiated state, the expression was minimal in stromal cells but strong in the epithelium, in which Spry-1 is distributed throughout the cytoplasm of basal and luminal cells (Fig. S3E, F).

### The Distribution of Decorin, SPARC, Spry-1 and Tsukushi in Human Foetal Prostate

**Figure 4:**
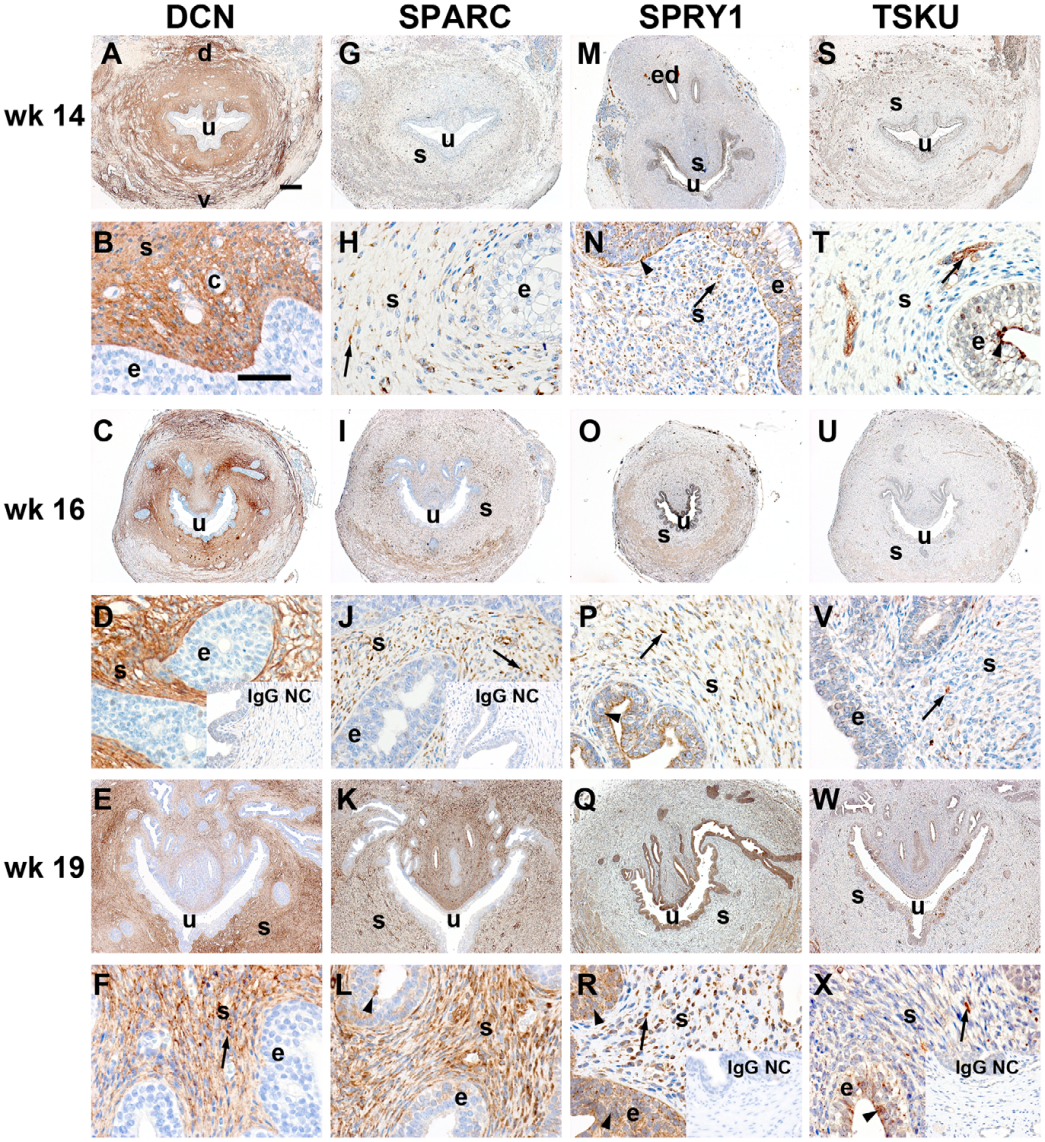
Decorin, SPARC, SPRY-1 and TSKU protein expression in human foetal prostates at gestational weeks 14 to 19. Human foetal prostates at gestational weeks 14, 16 and 19 are shown in the top two, middle two and bottom two rows, respectively (each age stage n = 1 tissue). The A–S, C–U and E–W rows represent overviews (scale bar  = 200 um), while the B–T, D–V and F–X rows show magnifications (scale bar  = 50 µm). Each column shows the staining for one protein across the stated developmental time period, and insets show IgG negative controls. Only Decorin expression was restricted to the mesenchyme, while SPARC and TSKU were also mesenchymal with a little epithelial expression at wk 19. SPRY-1 showed expression in mesenchyme and some epithelial expression with increasing age. Arrows indicated examples of mesenchymal expression while arrowheads indicate epithelial expression. Legend: d dorsal, e epithelium, ed efferent duct, s stroma, u urethra, v ventral.

Next, we examined the protein localisation of Decorin, SPARC, Spry-1 and Tsukushi in human foetal prostate. A wk 14 human prostate shows early epithelial budding, similar to rat perinatal stage P0.5 [21]; see also Fig. 4, two top rows). However, precise correlation of developmental stages is difficult due to anatomical differences, as rodents show paired and discrete lobes while the human prostate is compact without lobular organization [21].

Decorin expression in human foetal prostates was confined to the mesenchymal compartment and was completely absent in the urothelium and prostatic epithelium at all developmental stages (Fig. 4A–F). Notably, Decorin expression in the ventral smooth muscle areas was extremely low or absent (e.g. Fig. 4C, bottom of panel).

We were unable to localise Semaphorin6D in human foetal prostate due to poor antibody reactivity to human Sema6D (data not shown).

SPARC expression showed stronger staining with increasing foetal age and was predominantly expressed in the mesenchyme (Fig. 4G–L). At wk14, expression was adjacent to endothelial cells and was also present in smooth muscle at wk16 (Fig. 4K–L). At both stages, the epithelium was devoid of SPARC expression. At wk19, stromal staining was intense and found in most mesenchymal cells (Fig. 4K–L), including endothelial capillary cells, and with weak staining in the epithelia. The staining in the apical region of epithelial suggests that the SPARC epithelial signal may be non-specific (arrowhead in Fig. 4L) as prostate secretory proteins can non-specifically bind some antibodies.

Spry-1 was found to be expressed in both, the epithelial and mesenchymal compartments at all three ages (Fig. 4M–R). At wk14, the epithelial localisation was at the basal membrane and on top of the apical side of the luminal epithelial cells. Mesenchymal expression was confined to small but intense spots that were scattered throughout the mesenchyme (Fig. 4K–L). At wk16, epithelial expression was absent from the basement membrane but still present at the apical surface while mesenchymal expression was localised to the cytoplasm of a subset of cells, including endothelial cells. At wk19, epithelial expression was evenly distributed throughout the basal and luminal cell layers, localised to the cytoplasm and cell surface. Mesenchymal expression was once again confined to small spots within a subset of fibroblastic and endothelial cells.

Tsukushi was found in small mesenchymal subregions compared with the other three proteins, and its prevalence was minimal at all three stages (Fig. 4S–X). There was no clear expression pattern in regard to cell type or area, instead it was found in fibroblasts and endothelial cells, and was detected on the apical side of some luminal epithelial cells at weeks 14, 16 and 19.

Decorin, SPARC, Spry-1 and Tsukushi showed striking similarities in regard to mesenchymal localisation as well as increase in staining intensity in both human and rodent, between P0.5 to P6.5 and wk14 to wk19, respectively. The similarities in protein distribution between rat and human suggest that these molecules are mesenchymally expressed and may play important roles in developmental growth and patterning of the prostate.

### Expression of Decorin is Decreased in Prostate Cancer

Decorin and Tsukushi demonstrated the most mesenchyme-specific expression patterns in developing rat and human prostate, while Semaphorin6D, SPARC and Spry-1 showed both mesenchymal and epithelial expression. We decided to focus on molecules showing mesenchyme-only expression and thus Decorin and Tsukushi were taken forward for evaluation of expression in prostate cancer and patient-matched controls.

Tissue micro arrays (TMAs) and samples of our own collection of PCa were stained for Tsukushi but no difference was observed between PCa and non-malignant tissue (data not shown). Next, we examined Decorin expression.

**Figure 5:**
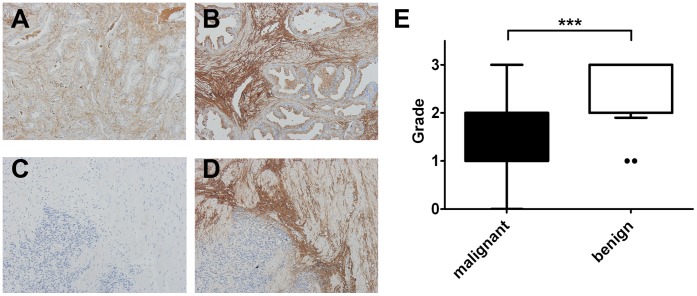
Decorin expression is decreased in prostate carcinomas. PCa tissue was stained via IHC for Decorin. A) A representative PCa tissue spot showed less intense staining than B) a representative non-malignant tissue spot. Simultaneously processed prostate tissue from our own tissue bank was used with non-specific IgG as negative control C) and anti-Decorin antibody as positive control (D), demonstrating specificity of staining. The evaluation of DAB staining intensity for each tissue spot is shown in E), demonstrating a significantly lower scoring in PCa (n = 91) compared with non-malignant controls (n = 20) (Mann Whitney test, two-tailed, p<0.0001). The boxes indicate the 25–75 percentiles while whiskers indicate the 10–90 percentiles.

Representative areas of Decorin staining are shown in Fig. 5A, B. Decorin staining was scored by a single blinded investigator for the extent of stromal Decorin staining (0 =  no staining, 1 = ∼1–20%, 2 = ∼20–50%, 3 = >50% of stromal area). Also, the most prevalent intensity of staining in each tissue spot was assessed (independent of the stained area size) with a non-linear scoring system 0,1,2,3 with 0 being absence of staining, and 1, 2, and 3 representing increasing stain intensity. We assumed that the staining intensity was in proportion to the actual amount of Decorin protein. Between malignant and non-malignant tissue, there was no difference in the extent of stromal area of Decorin expression (Mann-Whitney test; p = 0.45). However, we found a decreased intensity of Decorin staining in malignant areas compared with non-malignant control areas. In total we analysed 186 tissue spots from commercial TMAs and tissue sections of TURP chips from 10 patients.

Statistical analysis with a non-parametric Mann-Whitney test confirmed a significant decrease of intensity of stromal Decorin staining in malignant areas compared with non-malignant control areas (p = 0.0001, Fig. 5E). The results suggest a downregulation of Decorin in PCa. We next examined a possible correlation between Decorin staining intensity and Gleason score, however there was no evidence of correlation (data not shown).

**Table 2:** Primer sequences for the whole mount in situ experiments.

Oligo name	Sequence (5′ to 3′ orientation)	Product size [bp]
Dcn F	CGAAGACACATCTGAAGGTG	523
Dcn R	CAGTCAACTGCATCTGGATG	
Sema6D F	TTCTGCCACAGTGGCTGATT	548
Sema6D R	GCCTTGGTTTTGGTACTTTG	
SPARC F	CCCGAGACTTTGAGAAGAAC	518
SPARC R	AGGCTGTGGATAGGCTATGG	
Spry-1 F	CAAGCCGTCATGACTTCTGG	535
Spry-1 R	GTGAATCCAGAGCTGTGTGC	
Tsku F	CCAAGCTCAAGTGGGCAG	503
Tsku R	GCATCGAAGTCCCTTTGC	

## Discussion

Molecules expressed in prostate mesenchyme and stroma play key roles in development, tissue homeostasis and diseases such as cancer. We set out to identify molecules that showed mesenchyme-specific expression in the prostate, and subsequently those that also showed dysregulated expression in prostate cancer. Our starting point was a developmental gene expression profiling study from our group which provided a list of 219 molecules that were potentially restricted to inductive prostate mesenchyme. The transcript distribution of several of these candidates was examined by wholemount in situ hybridization, leading to the identification of five mesenchymally expressed molecules during prostate development. One of these molecules, the putative tumour suppressor Decorin, also showed downregulation in prostate cancer stroma.

**Table 3:** Antibodies for Immunohistochemistry.

Antibody	Raised in	Manufacturer/provider	Cat no.	Lot no.	final concentration (mg/ml)
Decorin	rabbit	Sigma-Aldrich	HPA003315	A08795	0.150
Semaphorin6D	rabbit	AbCam	Ab81255	831079	6.7
SPARC	rabbit	Santa Cruz	sc-25574	F1505	2.0
Sprouty1	mouse	Sigma-Aldrich	WH0010252M1	08193-3H4	3.3
Tsukushi	rabbit	Sigma-Aldrich	HPA008164	R02536	1.2
IgG fraction	rabbit	Dako	X0903	00029041	0.15 to 6.7*
IgG 2ak	mouse	Sigma-Aldrich	M5409	NA	3.3

* depending on concentration of primary antibody.

### Expression of Mesenchymal Molecules in Developing Prostates

We have identified mesenchymal expression of *Decorin, Semaphorin6D, SPARC, Sprouty1 and Tsukushi* in perinatal P0.5 male rat UGTs via WISH and their respective proteins via IHC. In all cases, there was a very good correlation between WISH staining and IHC detection of the molecules. The distribution of Decorin protein showed peri-epithelial localisation that was not detected via WISH. The Decorin antibody was validated in the Human Protein Atlas Project (http://www.proteinatlas.org/) as specific for human Decorin and as it is a secreted protein, we speculate that the strong staining adjacent to the urothelium is the result of secretion and diffusion.

*Decorin, Semaphorin6D, SPARC, Sprouty1* and *Tsukushi* were expressed in rat mesenchyme during E17.5 to P0.5. At P6.5 four molecules showed mesenchymal-only expression with only Spry-1 showing expression in both mesenchyme and epithelium. When prostate differentiation was nearly or fully complete at P28 and adult, respectively, there were clear differences between groups: the proteoglycans Decorin and Tsukushi had stromal localisation, Semaphorin6D gained a weak expression in the epithelium but was nearly completely absent in the stroma, whereas Spry-1 and SPARC showed a reversal of patterns with strong expression towards the epithelial compartment and near absence in the stroma. From these data we conclude that amongst the currently investigated molecules two retain stromal expression in the differentiated prostate and the remainder either cease stromal expression or switch to epithelial expression.

### Mesenchymal Expression in Rat and Human Prostate Development

There are recent reviews of comparative anatomy between human and rodent [21] as well as comparisons of global gene expression programmes in prostate development versus cancer [22]. However, few studies so far compared mesenchymally expressed genes and their protein products between the developing rodent and human prostate [23]. In the current study we found that the expression of Decorin, SPARC, Spry-1 and Tsukushi in human prostate correlated well with the respective developmental stages in the rat prostate, suggesting that the rat is a suitable model for the study of human prostate development.

### A Role for Decorin in Prostate Development and Cancer

Several functions have been described previously for Decorin in development. Decorin is a small connective tissue proteoglycan demonstrated to have multiple physiological functions including: structural function, context-dependent inhibition or enhancement of TGFβ signalling, restricting mitosis, and other activities [24]–[27]. Various studies have shown that TGFβ is important in prostate organogenesis and can enhance the mitotic rate in undifferentiated or less well differentiated epithelial cells at the tips of budding prostate lobes in the rat [28]. We speculate that the action of TGFβ can be modulated by Decorin, as described during lung branching where Decorin prevents the inhibitory activity of TGFβ in organ explants in vitro [29].

We identified Decorin as a stromally expressed molecule and examined if it showed altered expression in prostate cancer (PCa) stroma. In a PCa TMA we identified a reduction of Decorin staining compared with non-malignant controls. This result differs from a previous study that reported an increase in Decorin expression in early stage prostate cancers [30]. It is possible that the use of different antibodies and the relatively small number of samples investigated in both studies could contribute to the conflicting results. However, our finding of Decorin down-regulation in PCa is supported by its activity as a tumour suppressor in prostate cancer [31], [32] and further investigation of Decorin in prostate cancer is likely to be of value.

### What is the Potential Role of Decorin in Prostate Cancer?

Decorin exerts pleiotropic effects in tumour suppression, affecting multiple mechanisms and pathways.

In a model of colorectal cancer, Decorin was reported to interact with and stabilise E-cadherin, thereby attenuating the progression of colorectal cancer [33]. In another model system, Decorin led to an acidification of the microenvironment and cancer cells, resulting in the inhibition of migrating melanoma cells in a co-culture system with fibroblasts [34]. Regarding specific pathways, insulin-like growth factors (IGFs) are commonly upregulated in cancers and activate IGF1R, which subsequently activates the pro-tumourigenic PI3K-Akt pathway. Iozzo et al. found Decorin to antagonise the action of IGF1R in bladder cancer cells, which was in agreement with the findings that Decorin was decreased at the mRNA and protein level in bladder cancer tissue of patient samples [35].

Several of these diverse mechanisms could be relevant to prostate cancer. Since the genes for glucose metabolism are highly overexpressed in several cancers, including prostate, this indicates the presence of the Warburg effect [36], which ultimately results in acidosis. Hence, Decorin as main contributor to acidification of the microenvironment appears unlikely in PCa, as it appears less abundant in the prostate tumour stroma.

The idea of Decorin stabilising E-Cadherin appears unlikely for the prostate, as Decorin is not expressed in prostate epithelial cells. Also, previous reports showed that the most aggressive forms of prostate cancer metastasis have the highest E-Cadherin expression [37]. As such, the potential interaction between E-Cadherin and Decorin in PCa is speculative.

Generally, the role of IGF1 and its cognate receptor IGF1-R are well established to participate in tumourigenesis [38]. In regard to PCa, there are bioinformatic studies demonstrating a correlation between IGF1 levels and the onset of PCa. In one study, IGF1 was identified as one of the top ten genes probable to drive prostate cancer [39]. Another group found that IGF1 is likely to be secreted from normal genomically stable cells towards the cancer cells, thereby acting in a paracrine manner [40]. Inhibition of IGF1-R via chemical compounds had inhibitory effects in vitro on PCa colony formation in a 3D matrix [41]. Therefore we speculate that studies into the interaction of Decorin and IGF1 signalling could yield a better mechanistic understanding of PCa inhibition via Decorin.

Furthermore, activated cancer-associated fibroblasts (CAF) are part of the tumour microenvironment and well-known to support tumour growth [7]. It was found that they secrete higher levels of TGFβ than morphologically normal prostatic fibroblasts [10]. TGFβ acts as an autocrine signal to maintain CAF properties and additionally signals to the neighbouring cells. It stimulates cancer growth and angiogenesis in a paracrine manner as well as further myo-differentiation of fibroblasts and thereby supports an overall disease progression [42]–[45]. Hence, we speculate that Decorin might interfere –among other things- with TGFβ action and exogenously administered Decorin protein could have potential beneficial therapeutic effects.

### Summary

In this study we describe detailed spatial-temporal expression of Decorin, Semaphorin6D, SPARC, Sprouty1 & Tsukushi in developing rat and human prostate. The expression patterns showed evolutionary conservation emphasising the importance of stromally restricted factors. We identified Decorin as a stromally restricted molecule whose expression was down-regulated in prostate cancer. These findings confirm the concept that developmental mesenchymal molecules are important to human diseases such as prostate cancer. Further studies into Decorin’s potentially tumour suppressive effects in prostate cancer are warranted.

## Materials and Methods

### Ethical Statements

The use of human foetal reproductive tissues from medical terminations of pregnancies was approved by the Lothian Research Ethics Committee (study code LREC 08/S1101/1). All participants gave informed written consent according to UK national guidelines.

Patients undergoing transurethral resection of the prostate (TURP) provided informed written consent. The use of their TURP tissue was approved by the Eastern Multicentre Research Ethics Committee, Cambridge, reference no. MREC 02/5/63.

The animal procedures included breeding & euthanising and were carried out by trained staff to optimise animal health & minimise suffering, respectively. Euthanisation was performed in accordance with ‘schedule 1’, defined by the UK Animal Scientific Procedures Act (1986).

### Animals

The Wistar rat strain is an established model for investigating prostate development. Wistar rats were housed under standard conditions with 12 h/12 h light dark cycles and *ad libidum* access to water and food. Animals were euthanised by cervical dislocation and/or decapitation for organ dissection afterwards. Tissues were micro-dissected from the urogenital tract (UGT) for which the copulatory plug observation was taken as embryonic day 0.5 (E0.5), and the day of birth was designated P0.5.

### Human Tissue

Gestational stage was determined by ultrasound scan and confirmed by subsequent direct measurement of foot length. One prostate each of 14, 16 and 19 weeks of gestation were dissected into sterile PBS solution before fixation in Bouin’s solution on ice for two hours.

Prostate cancer tissue was collected at the Western General Hospital, Edinburgh, Scotland. 10 of the 34 collected samples originated from patients naïve to previous PCa-treatment and their formalin-fixed prostate tissue samples were used for immunohistochemistry. Their pathology details are given in Tables S1, S2, S3.

Two commercial PCa TMAs were purchased from AccuMax Array: TMA A222(II), lot 12210505231, contained malignant samples in duplicates from 45 patients of which 6 were also used to provide single-spot non-malignant control tissue. TMA A222(III), lot 1221120707312, contained 45 PCa patient samples in duplicates and provided 8 single control spots. The TMAs’ accompanying pathological data are described in Tables S2 and S3. In addition, we used our own collection of PCa samples and identified 10 patients that did not receive therapy prior to TURP and hence had preserved tissue architecture (for pathological details see Table S1). 74 TURP chips of the ten patients were pathologically evaluated for malignant and non-malignant areas in the first and last slide in a row of serial sections, from which slides in between were used for Decorin staining. Up to two randomly chosen fields of malignant and non-malignant areas each were imaged and evaluated for Decorin staining.

### Profiling Data

The tag profiling data were collected in a previous study [17] and are accessible from the Gene Expression Omnibus (GEO) data base under accession number GSE7899.

### Whole Mount in Situ Hybridization (WISH)

Generation of DIG-labelled sense and antisense RNA probes was undertaken using methods detailed previously [19]. The sequences of the primers were checked via BLAST for target-specificity and are given in Table 2. Tissue-specificity was checked by normal RT-PCR and subsequent confirmation of expected band size via agarose gel electrophoresis of PCR products.

P0.5 urogenital tracts (n = 50) were micro-dissected and fixed overnight in 4% paraformaldehyde, dehydrated through graded methanol, and stored in 100% methanol at −20°C; RNA in situ hybridization was performed using the InsituPro VS robot (Intavis Bioanalytical Instruments, AG, Cologne, Germany), using a previously published protocol [19]. In short, hybridization with DIG-labelled probes was performed at 65°C for 16 h, followed by high stringency washes. For detection, tissues were incubated with anti-DIG antibody conjugated to alkaline phosphatase (1∶2000; Roche) at 18°C for 16 h. After washing, the colour was developed using Nitro-Blue Tetrazolium Chloride/5-Bromo-4-Chloro-3′-Indolyphosphate p-Toluidine Salt solution (Roche). Typical development times were around 8 h. Digital images were acquired with a Leica MZ6 stereo microscope (Leica Microsystems, Deerfield, IL) with attached Leica ICA camera.

### Immunohistochemistry

Immunolocalisation of candidates on rat and human tissues was undertaken using specific antibodies and appropriate IgG negative controls (Table 3). Rat tissues were obtained at E17.5 (n = 3 litters), E19.5 (n = 4 litters), P0.5 (n = 15 litters), P6.5 (n = 6), P28 (n = 4) and adults (3 months, n = 5). Tissues were fixed for two hours on ice in Bouin’s, paraffin-embedded, sectioned at 5 microns and mounted onto frosted slides. Tissue slides were dewaxed and rehydrated in a xylene/alcohol series, and antigens retrieved via pressure cooking the sections in 0.01 M citrate buffer, pH 6. The antigen detection and DAB staining was performed on a Bond-Max robot (Leica Microsystems, Peterborough, UK) with the Bond Polymer Refine Detection Kit (cat. No. DS9800; Vision BioSystems bond, Newcastle upon Tyne, UK) according to the manufacturer’s instructions. After the run, slides were dehydrated in an alcohol/xylene series, mounted with Pertex (Histolab, Gothenburg, Sweden) and cover slipped. Images were taken with an Axiocam HRc digital camera (Zeiss, Welwyn Garden City, UK) mounted on a Provis AX70 microscope (Olympus, Essex, UK). Images were moderately white balanced without any loss of information, assembled and annotated with Photoshop.

#### Statistical analysis

Data were analysed with GraphPadPrism5 (GraphPad Software Inc., La Jolla, CA, USA) and statistical tests were chosen as appropriate and indicated in the text.

## Supporting Information

Figure S1 **Immunolocalisation of Semaphorin6D in the developing rat prostate.** Developmental stages of E17.5 (A), E19.5 (B), P0.5 (C), P6.5 (D) P28 (not shown, as it looks like adult) and young adult VP (E) were investigated. At developmental stages E17.5, E19.5 the magnifications show the areas of prospective VP (also indicated by arrows in A, B), while at P0.5 and P6.5 it is either the VP or the DP. Semaphorin6D was absent in any epithelium and expressed only in mesenchyme from E17.5 until P6.5 (arrows in A–D). However, at P28 and adult stages, it was also found in epithelial cells and macrophages (arrowheads in E). Note predominant localisation to the nuclei. Scale bar equals 200 um in A and all other panels are at the same scale.
(TIF)

Figure S2 **Immunolocalisation of SPARC in the developing rat prostate.** Developmental stages of E17.5 and E19.5 did not show positive staining (data not shown). Stages P0.5 (A), P6.5 (B), P28 (C) and young adult VP (D) are shown. At developmental stages P0.5 and P6.5 the magnifications show the areas of either VP or DP. SPARC showed weak mesenchymal-only expression at P0.5 and P6.5, localised to the cytoplasm (arrows in A, B). However, at P28 and adult stages, there was strong staining in the nuclei of a subset of epithelial cells and macrophages (arrowheads in C, D), while stromal expression was very weak or absent. Scale bar equals 200 um in A, and all other panels are at the same scale.
(TIF)

Figure S3 **Immunolocalisation of Spry-1 in the developing rat prostate.** Developmental stages of E17.5 (A), E19.5 (B), P0.5 (C), P6.5 (D), P28 (E) and young adult VP (F) were investigated. At developmental stages E17.5, E19.5 the magnifications show the areas of prospective VP, while at P0.5 and P6.5 it is either the VP or the DP. Sprouty1 protein localisation was mesenchymal only at E17.5 and E19.5 (arrows). At P0.5, the expression was strong in the mesenchyme that surrounds epithelial ducts, with very weak expression in the epithelium. At P6.5, the epithelial expression was stronger than at P0.5 but still much weaker than in the mesenchyme. At P28, the expression was reversed: strong in the epithelium (arrowheads in E, F), weak in the stroma; and also in adult VP. Scale bar equals 200 um in A, and all other panels are at the same scale.
(TIF)

Table S1 **Pathological details of pre-treatment-naïve PCa patients undergoing transurethral resection of the prostate.**
(XLS)

Table S2 **Pathological details of PCa patients in the tissue micro array A222(II).**
(XLS)

Table S3 **Pathological details of PCa patients in the tissue micro array A222(III).**
(XLS)
