# Supplementary figures and images for: Stromal Expression of Decorin, Semaphorin6D, SPARC, Sprouty1 and Tsukushi in Developing Prostate and Decreased Levels of Decorin in Prostate Cancer
Source: PLoS One. 2012 Aug 3;7(8):e42516. doi: 10.1371/journal.pone.0042516 (PMC3411755; doi:10.1371/journal.pone.0042516)

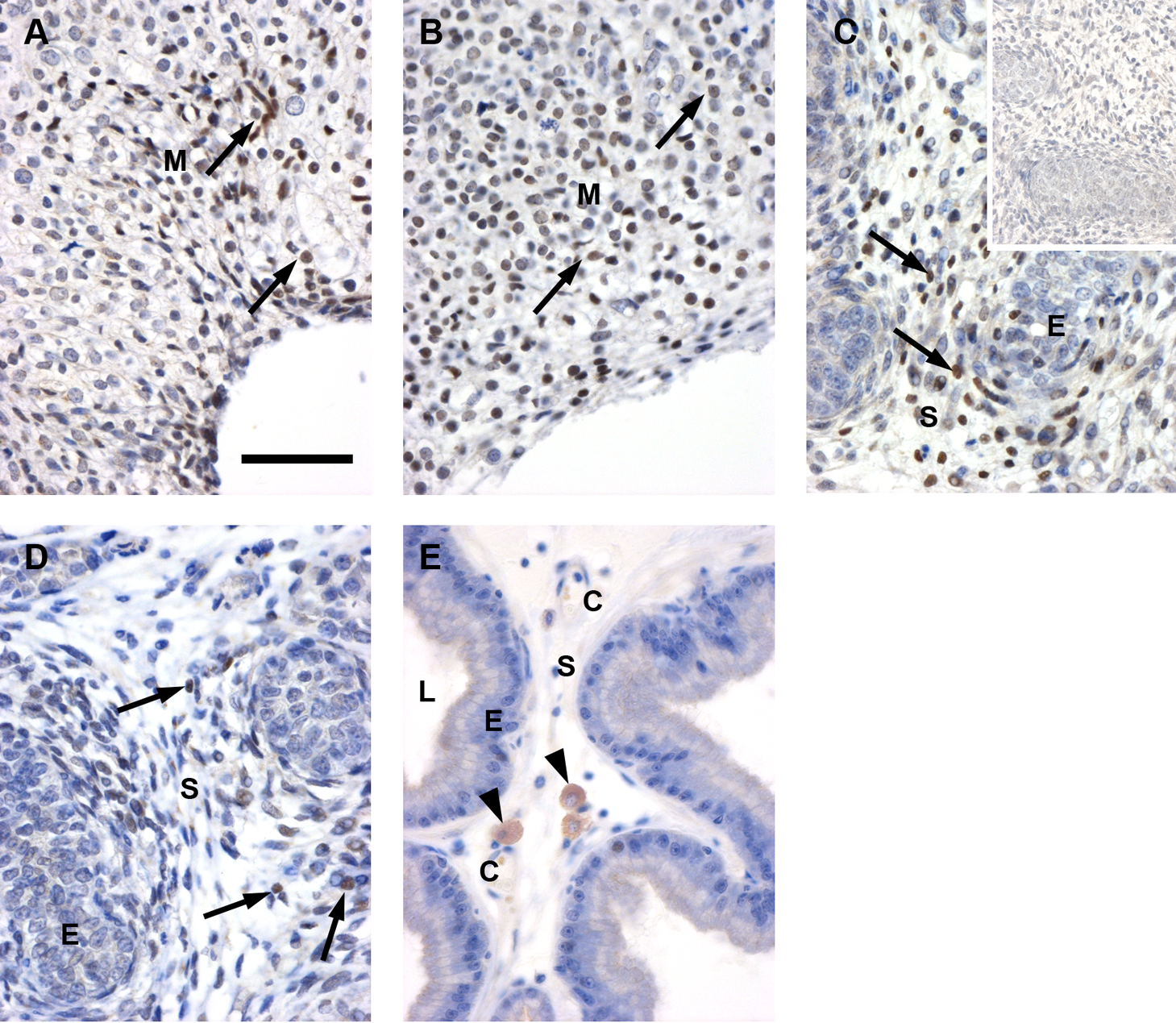

Supplement: Figure S1 — Immunolocalisation of Semaphorin6D in the developing rat prostate. Developmental stages of E17.5 (A), E19.5 (B), P0.5 (C), P6.5 (D) P28 (not shown, as it looks like adult) and young adult VP (E) were investigated. At developmental stages E17.5, E19.5 the magnifications show the areas of prospective VP (also indicated by arrows in A, B), while at P0.5 and P6.5 it is either the VP or the DP. Semaphorin6D was absent in any epithelium and expressed only in mesenchyme from E17.5 until P6.5 (arrows in A–D). However, at P28 and adult stages, it was also found in epithelial cells and macrophages (arrowheads in E). Note predominant localisation to the nuclei. Scale bar equals 200 um in A and all other panels are at the same scale. (TIF) [file pone.0042516.s001.tif]

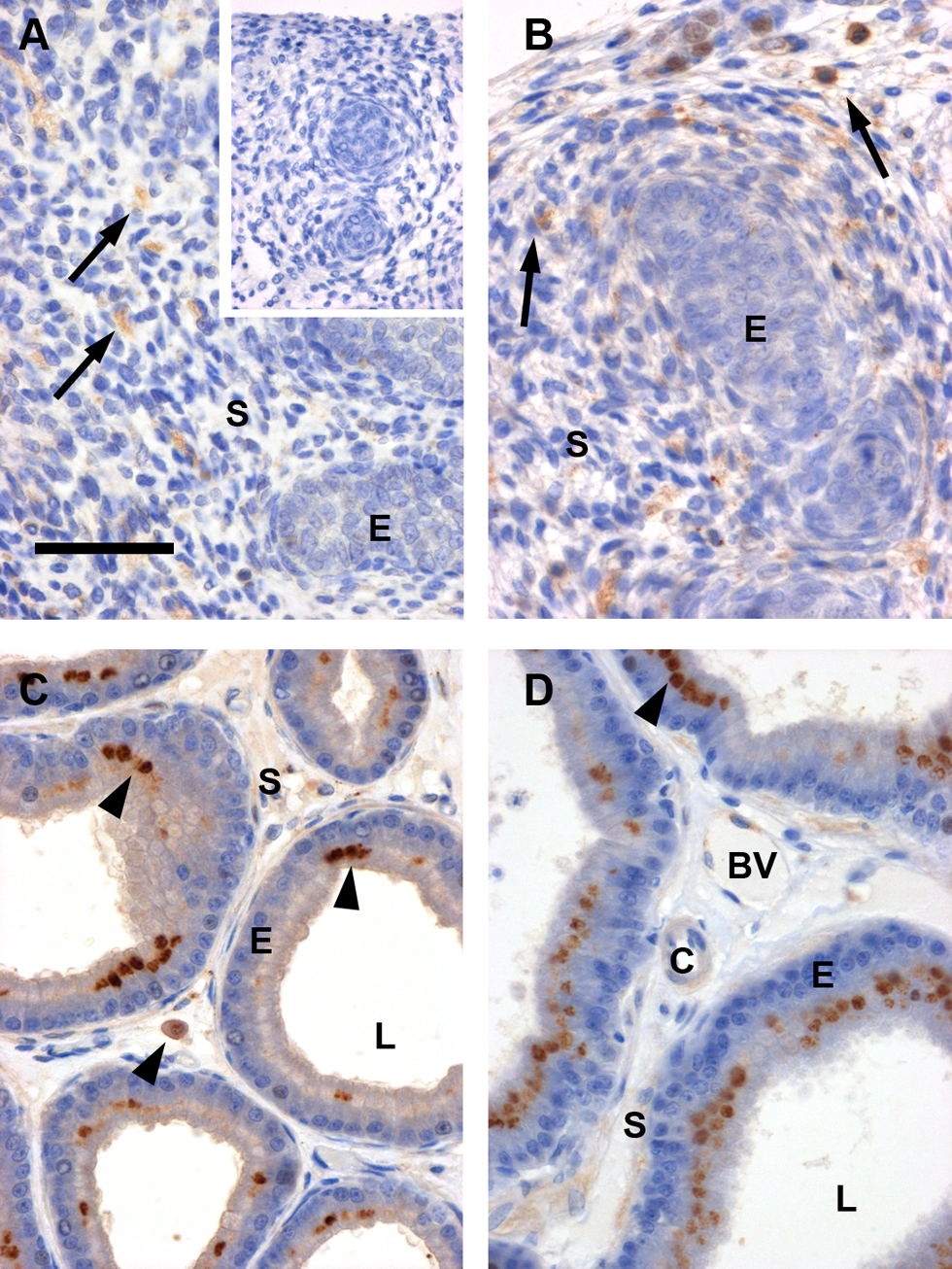

Supplement: Figure S2 — Immunolocalisation of SPARC in the developing rat prostate. Developmental stages of E17.5 and E19.5 did not show positive staining (data not shown). Stages P0.5 (A), P6.5 (B), P28 (C) and young adult VP (D) are shown. At developmental stages P0.5 and P6.5 the magnifications show the areas of either VP or DP. SPARC showed weak mesenchymal-only expression at P0.5 and P6.5, localised to the cytoplasm (arrows in A, B). However, at P28 and adult stages, there was strong staining in the nuclei of a subset of epithelial cells and macrophages (arrowheads in C, D), while stromal expression was very weak or absent. Scale bar equals 200 um in A, and all other panels are at the same scale. (TIF) [file pone.0042516.s002.tif]

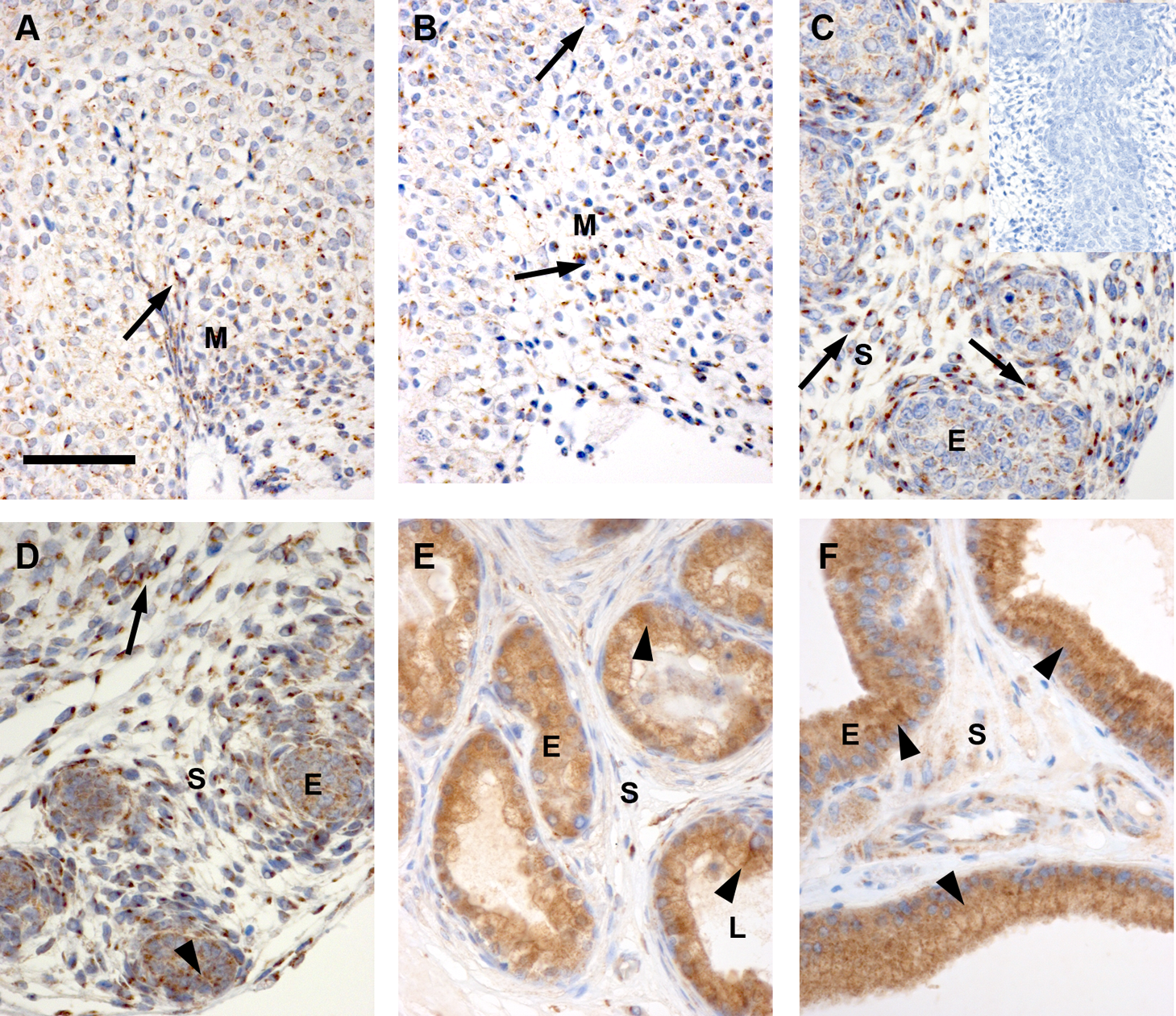

Supplement: Figure S3 — Immunolocalisation of Spry-1 in the developing rat prostate. Developmental stages of E17.5 (A), E19.5 (B), P0.5 (C), P6.5 (D), P28 (E) and young adult VP (F) were investigated. At developmental stages E17.5, E19.5 the magnifications show the areas of prospective VP, while at P0.5 and P6.5 it is either the VP or the DP. Sprouty1 protein localisation was mesenchymal only at E17.5 and E19.5 (arrows). At P0.5, the expression was strong in the mesenchyme that surrounds epithelial ducts, with very weak expression in the epithelium. At P6.5, the epithelial expression was stronger than at P0.5 but still much weaker than in the mesenchyme. At P28, the expression was reversed: strong in the epithelium (arrowheads in E, F), weak in the stroma; and also in adult VP. Scale bar equals 200 um in A, and all other panels are at the same scale. (TIF) [file pone.0042516.s003.tif]
